# (1*R*,4*S*,8*R*,9*R*,12*S*,13*S*,14*R*,16*S*,17*R*,19*R*)-17-[(Ethyl­sulfan­yl)meth­yl]-9,14-di­hydroxy-7,7-dimethyl-2,18-dioxo-3,10-dioxapenta­cyclo[14.2.1.0^1,13^.0^4,12^.0^8,12^]nona­decan-19-yl acetate acetone solvate

**DOI:** 10.1107/S1600536808043341

**Published:** 2008-12-24

**Authors:** Hao Shi, Hong Xiang Sun

**Affiliations:** a The College of Pharmaceutical Science, Zhejiang University of Technology, Hangzhou 310014, People’s Republic of China; b College of Animal Sciences, Zhejiang University, Hangzhou 310029, People’s Republic of China

## Abstract

The title compound, C_24_H_32_O_8_S·C_3_H_6_O, features three six-membered and two five-membered rings. The six-membered rings adopt chair, boat and slightly distorted boat conformations whereas one five-membered ring adopts an approximate envelope conformation and the other a twist conformation. Disorder was modelled for the ethyl­thio group with the ethyl-C atoms resolved over three positions with occupancies of 0.58 (4), 0.23 (4) and 0.19 (3). In the crystal, an O—H⋯O hydrogen bond links the molecules into chains.

## Related literature

For puckering parameters, see: Cremer & Pople (1975 ▶). For related literature, see: Yamaguchi *et al.* (1977 ▶); Chen *et al.* (1987 ▶); He *et al.* (2007 ▶); Shi *et al.* (2007 ▶).
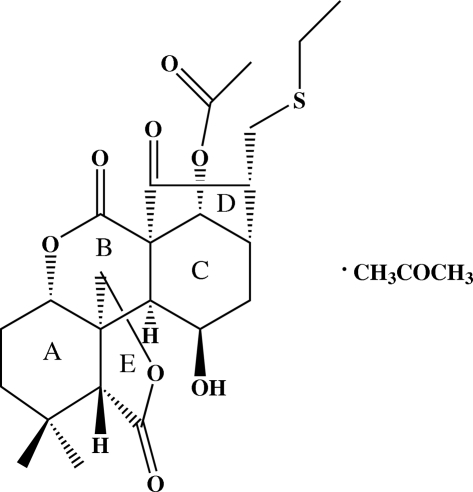


## Experimental

### 

#### Crystal data

C_24_H_32_O_8_S·C_3_H_6_O
*M* *_r_* = 538.63
Orthorhombic, 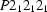

*a* = 10.6258 (12) Å
*b* = 11.4825 (18) Å
*c* = 22.265 (2) Å
*V* = 2716.6 (6) Å^3^
*Z* = 4
Mo *K*α radiation
μ = 0.17 mm^−1^
*T* = 298 (2) K
0.53 × 0.32 × 0.28 mm

#### Data collection

Bruker SMART CCD area-detector diffractometer
Absorption correction: multi-scan (*SADABS*; Bruker, 1999 ▶) *T*_min_ = 0.915, *T*_max_ = 0.954
11871 measured reflections
4774 independent reflections
3201 reflections with *I* > 2σ(*I*)
*R*_int_ = 0.041

#### Refinement

*R*[*F* ^2^ > 2σ(*F* ^2^)] = 0.044
*wR*(*F* ^2^) = 0.112
*S* = 1.04
4774 reflections
373 parameters
1 restraint
H-atom parameters constrained
Δρ_max_ = 0.31 e Å^−3^
Δρ_min_ = −0.23 e Å^−3^
Absolute structure: Flack (1983 ▶), 2053 Friedel pairs
Flack parameter: 0.00 (12)

### 

Data collection: *SMART* (Bruker, 1999 ▶); cell refinement: *SAINT* (Bruker, 1999 ▶); data reduction: *SAINT*; program(s) used to solve structure: *SHELXS97* (Sheldrick, 2008 ▶); program(s) used to refine structure: *SHELXL97* (Sheldrick, 2008 ▶); molecular graphics: *ORTEP-3* (Farrugia, 1997 ▶); software used to prepare material for publication: *SHELXL97*.

## Supplementary Material

Crystal structure: contains datablocks I, global. DOI: 10.1107/S1600536808043341/tk2336sup1.cif

Structure factors: contains datablocks I. DOI: 10.1107/S1600536808043341/tk2336Isup2.hkl

Additional supplementary materials:  crystallographic information; 3D view; checkCIF report

## Figures and Tables

**Table 1:** Hydrogen-bond geometry (Å, °)

*D*—H⋯*A*	*D*—H	H⋯*A*	*D*⋯*A*	*D*—H⋯*A*
O5—H5⋯O8^i^	0.82	2.16	2.952 (4)	161

Symmetry code: (i) 
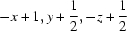
.

**Fig. 1.**
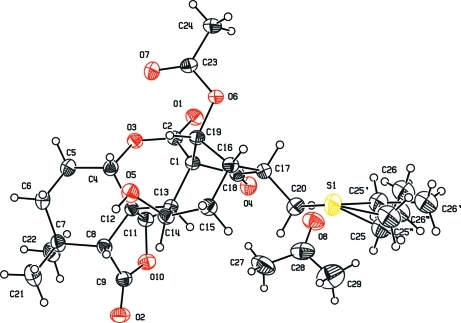
The structure of (1) showing atomic numbering scheme and 30% probability displacement ellipsoids. The ethylthio group is disordered over three positions.

**Fig. 2.**
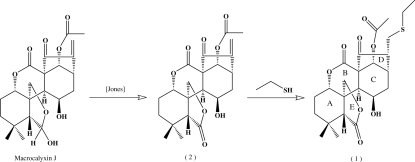
Reaction scheme.

## supplementary crystallographic information

### Comment

Since the natural product diterpenoid Macrocalyxin J exhibits cytotoxicity against cultures of Hela cells (Shi *et al.*, 2007), the title compound (1), a derivative, was synthesised for investigation. The molecule of (1) is composed of three six-membered and two five-membered rings, Fig. 1. The cyclohexane ring A (C4—C8/C12) adopts a chair conformation with puckering parameters (Cremer & Pople, 1975) Q = 0.525 (4) Å, θ = 156.0 (4)° and φ = 275.2 (10)°. Ring B (O3/C2/C1/C13/C12/C4) exists in a distorted boat conformation (Q = 0.671 (3) Å, θ = 109.0 (3)° and φ = 94.9 (3)°) and ring C (C1/C13—C16/C19) adopts a boat conformation (Q = 0.851 (3) Å, θ = 79.9 (2) and φ = 296.0 (2)°). The five-membered ring D (C1/C18/C17/C16/C19) is twisted on C(16)/C(19), and five-membered ring E (O10/C9/C8/C12/C11) adopts an envelope conformation with C(12) displaced by 0.500 (5)Å from the mean plane of the remaining four atoms. The stereochemistry of the A/B ring juncture is *trans*, and at the B/C ring juncture, *cis*. With the C1, C19 and C16 atoms being located in both rings C and D, an α-configuration is adopted to avoid steric crowding; no evidence was found in this study for the synthesis of the β-configuration. The main difference between (1) and macrocalyxin J (He *et al.*, 2007) is found in ring D. In the latter, the equivalent ring D is conjugated, i.e. is a α-methylenecyclopentanone ring. It has been reported that the α-methylenecyclopentanone group in the Rabdosia diterpenes is highly reactive toward sulfhydryl groups, essential to enzyme function (Yamaguchi *et al.*, 1977). This observation is ascribed to the steric strain within the five-membered ring which increases the reactivity of the conjugated double bond (Chen *et al.*, 1987). Compound (1) was characterised as an acetone solvate. In the crystal structure the constituent molecules are connected via hydrogen bonding, Table 1.

### Experimental

Compound (1) was obtained in a two-step syntheses, Scheme 1. In an ice-water bath, Jones reagent (0.2 ml ) was added to a solution of Macrocalyxin J (200 mg; isolated from Rabdosia macrocalyx) in acetone (20 ml). After stirring for 20 min, the solution was filtered and added to 15% NaHCO_3_ in water (120 ml). The mixture was extracted 3 times with diethyl ether (90 ml). After evaporation of the solvent, a white residue was isolated. Recrystallization from methanol gave 19-acetyloxy-10,13-dideoxy-5-hydroxy-10-oxo, (5β,19*R*)-enmain (175.6 mg) (2), see Scheme. Into an ethanol solution (50 ml) of (2) (150 mg) was added dropwise excess ethanethiol (0.5 ml). After stirring at room temperature for 3 h, the mixture was concentrated *in vacuo* to give an oily residue, which was washed with water (2.0 ml), then recrystallized from CH_3_COCH_3_ to afford (1) as colorless crystals (127.3 mg).

### Refinement

All H atoms were placed in geometrically calculated positions, and allowed to ride on their parent atoms with O-H = 0.82 Å and C-H = 0.96 - 0.98 Å, and with U_iso_(H) = 1.2U_eq_(C) for methylene- and methine-H, and 1.5U_eq_ for other H atoms. The ethyl-C atoms of the ethylthio group were found to be disordered over three positions (C25/C26, C25'/C26', and C25"/C26") with refined occupancies of 0.58 (4), 0.19 (3) and 0.23 (4).

### Crystal data

**Table:**

C_24_H_32_O_8_S·C_3_H_6_O	*F*(000) = 1152
*M**_r_* = 538.63	*D*_x_ = 1.317 Mg m^−^^3^
Orthorhombic, *P*2_1_2_1_2_1_	Mo *K*α radiation, λ = 0.71073 Å
Hall symbol: P 2ac 2ab	Cell parameters from 3142 reflections
*a* = 10.6258 (12) Å	θ = 2.6–24.5°
*b* = 11.4825 (18) Å	µ = 0.17 mm^−^^1^
*c* = 22.265 (2) Å	*T* = 298 K
*V* = 2716.6 (6) Å^3^	Needle, colorless
*Z* = 4	0.53 × 0.32 × 0.28 mm

### Data collection

**Table:**

Bruker SMART CCD area-detector diffractometer	4774 independent reflections
Radiation source: fine-focus sealed tube	3201 reflections with *I* > 2σ(*I*)
graphite	*R*_int_ = 0.041
φ and ω scans	θ_max_ = 25.0°, θ_min_ = 1.8°
Absorption correction: multi-scan (*SADABS*; Bruker, 1999)	*h* = −12→11
*T*_min_ = 0.915, *T*_max_ = 0.954	*k* = −13→13
11871 measured reflections	*l* = −23→26

### Refinement

**Table:**

Refinement on *F*^2^	Secondary atom site location: difference Fourier map
Least-squares matrix: full	Hydrogen site location: inferred from neighbouring sites
*R*[*F*^2^ > 2σ(*F*^2^)] = 0.044	H-atom parameters constrained
*wR*(*F*^2^) = 0.112	*w* = 1/[σ^2^(*F*_o_^2^) + (0.0474*P*)^2^ + 0.2952*P*] where *P* = (*F*_o_^2^ + 2*F*_c_^2^)/3
*S* = 1.04	(Δ/σ)_max_ = 0.001
4774 reflections	Δρ_max_ = 0.31 e Å^−^^3^
373 parameters	Δρ_min_ = −0.23 e Å^−^^3^
1 restraint	Absolute structure: Flack (1983), 2053 Friedel pairs
Primary atom site location: structure-invariant direct methods	Flack parameter: 0.00 (12)

### Special details

**Table:**

Geometry. All e.s.d.'s (except the e.s.d. in the dihedral angle between two l.s. planes) are estimated using the full covariance matrix. The cell e.s.d.'s are taken into account individually in the estimation of e.s.d.'s in distances, angles and torsion angles; correlations between e.s.d.'s in cell parameters are only used when they are defined by crystal symmetry. An approximate (isotropic) treatment of cell e.s.d.'s is used for estimating e.s.d.'s involving l.s. planes.
Refinement. Refinement of *F*^2^ against ALL reflections. The weighted *R*-factor *wR* and goodness of fit *S* are based on *F*^2^, conventional *R*-factors *R* are based on *F*, with *F* set to zero for negative *F*^2^. The threshold expression of *F*^2^ > σ(*F*^2^) is used only for calculating *R*-factors(gt) *etc*. and is not relevant to the choice of reflections for refinement. *R*-factors based on *F*^2^ are statistically about twice as large as those based on *F*, and *R*- factors based on ALL data will be even larger.

### Fractional atomic coordinates and isotropic or equivalent isotropic displacement parameters (Å^2^)

**Table:**

	*x*	*y*	*z*	*U*_iso_*/*U*_eq_	Occ. (<1)
S1	0.47821 (11)	0.18342 (10)	0.03744 (4)	0.0714 (3)	
O1	0.3800 (2)	−0.2350 (2)	0.24301 (10)	0.0512 (6)	
O2	0.5729 (2)	0.2605 (2)	0.40864 (11)	0.0619 (7)	
O3	0.3052 (2)	−0.14753 (19)	0.32251 (9)	0.0452 (6)	
O4	0.5666 (2)	−0.0665 (2)	0.19581 (10)	0.0529 (6)	
O5	0.1792 (2)	0.17561 (19)	0.27787 (9)	0.0503 (6)	
H5	0.1552	0.2387	0.2908	0.075*	
O6	0.2073 (2)	−0.10279 (18)	0.15674 (9)	0.0399 (5)	
O7	0.0895 (2)	−0.1727 (2)	0.23175 (10)	0.0564 (7)	
O8	0.8572 (3)	−0.1043 (3)	0.15591 (13)	0.0797 (9)	
O10	0.5889 (2)	0.0790 (2)	0.37478 (10)	0.0525 (6)	
C1	0.3555 (3)	−0.0293 (3)	0.23341 (13)	0.0358 (7)	
C2	0.3480 (3)	−0.1452 (3)	0.26595 (14)	0.0393 (8)	
C4	0.2686 (3)	−0.0359 (3)	0.34878 (14)	0.0432 (9)	
H4	0.1964	−0.0048	0.3266	0.052*	
C5	0.2298 (4)	−0.0579 (3)	0.41315 (15)	0.0564 (10)	
H5A	0.2961	−0.0990	0.4343	0.068*	
H5B	0.1542	−0.1051	0.4143	0.068*	
C6	0.2057 (4)	0.0590 (3)	0.44251 (16)	0.0594 (10)	
H6A	0.1704	0.0463	0.4821	0.071*	
H6B	0.1440	0.1013	0.4190	0.071*	
C7	0.3247 (3)	0.1332 (3)	0.44839 (14)	0.0491 (9)	
C8	0.3832 (3)	0.1511 (3)	0.38431 (13)	0.0425 (8)	
H8	0.3429	0.2181	0.3650	0.051*	
C9	0.5214 (3)	0.1744 (3)	0.39072 (14)	0.0454 (8)	
C11	0.5053 (3)	−0.0131 (3)	0.35640 (14)	0.0429 (8)	
H11A	0.4944	−0.0692	0.3886	0.051*	
H11B	0.5386	−0.0532	0.3215	0.051*	
C12	0.3793 (3)	0.0458 (3)	0.34119 (13)	0.0358 (7)	
C13	0.3838 (3)	0.0815 (2)	0.27396 (13)	0.0348 (7)	
H13	0.4713	0.1030	0.2658	0.042*	
C14	0.3048 (3)	0.1865 (3)	0.25581 (13)	0.0413 (8)	
H14	0.3424	0.2559	0.2741	0.050*	
C15	0.3051 (3)	0.2031 (3)	0.18730 (13)	0.0454 (8)	
H15A	0.2361	0.2542	0.1764	0.054*	
H15B	0.3828	0.2415	0.1758	0.054*	
C16	0.2929 (3)	0.0896 (3)	0.15153 (14)	0.0400 (8)	
H16	0.2374	0.1010	0.1169	0.048*	
C17	0.4186 (3)	0.0347 (3)	0.13140 (14)	0.0415 (8)	
H17	0.3984	−0.0246	0.1012	0.050*	
C18	0.4621 (3)	−0.0291 (3)	0.18731 (13)	0.0391 (8)	
C19	0.2390 (3)	−0.0040 (3)	0.19337 (13)	0.0365 (8)	
H19	0.1670	0.0244	0.2167	0.044*	
C20	0.5225 (4)	0.1088 (3)	0.10578 (15)	0.0566 (10)	
H20A	0.5948	0.0598	0.0976	0.068*	
H20B	0.5474	0.1660	0.1355	0.068*	
C21	0.2897 (4)	0.2544 (4)	0.47245 (16)	0.0684 (11)	
H21A	0.2283	0.2894	0.4465	0.103*	
H21B	0.3636	0.3025	0.4737	0.103*	
H21C	0.2554	0.2471	0.5122	0.103*	
C22	0.4149 (4)	0.0758 (3)	0.49357 (14)	0.0628 (11)	
H22A	0.3731	0.0668	0.5315	0.094*	
H22B	0.4879	0.1241	0.4986	0.094*	
H22C	0.4400	0.0008	0.4788	0.094*	
C23	0.1289 (3)	−0.1822 (3)	0.18166 (15)	0.0426 (8)	
C24	0.1034 (3)	−0.2786 (3)	0.13884 (15)	0.0548 (10)	
H24A	0.1809	−0.3173	0.1292	0.082*	
H24B	0.0667	−0.2476	0.1028	0.082*	
H24C	0.0463	−0.3331	0.1569	0.082*	
C25	0.624 (4)	0.193 (4)	−0.0035 (14)	0.077 (6)	0.58 (4)
H25A	0.6206	0.2573	−0.0320	0.092*	0.58 (4)
H25B	0.6935	0.2066	0.0238	0.092*	0.58 (4)
C26	0.640 (3)	0.0763 (19)	−0.0366 (13)	0.090 (5)	0.58 (4)
H26A	0.7155	0.0784	−0.0603	0.134*	0.58 (4)
H26B	0.5689	0.0632	−0.0624	0.134*	0.58 (4)
H26C	0.6458	0.0143	−0.0078	0.134*	0.58 (4)
C25'	0.607 (6)	0.117 (6)	−0.005 (2)	0.077 (14)	0.19 (3)
H25C	0.6823	0.1330	0.0182	0.092*	0.19 (3)
H25D	0.5931	0.0333	−0.0017	0.092*	0.19 (3)
C26'	0.641 (3)	0.139 (5)	−0.069 (2)	0.090 (15)	0.19 (3)
H26D	0.6900	0.0752	−0.0839	0.134*	0.19 (3)
H26E	0.6883	0.2097	−0.0717	0.134*	0.19 (3)
H26F	0.5651	0.1461	−0.0924	0.134*	0.19 (3)
C25"	0.596 (10)	0.185 (12)	−0.021 (4)	0.09 (2)	0.23 (4)
H25E	0.5544	0.1760	−0.0594	0.108*	0.23 (4)
H25F	0.6365	0.2610	−0.0210	0.108*	0.23 (4)
C26"	0.700 (7)	0.091 (4)	−0.016 (3)	0.090 (13)	0.23 (4)
H26G	0.7770	0.1210	−0.0330	0.134*	0.23 (4)
H26H	0.6747	0.0231	−0.0382	0.134*	0.23 (4)
H26I	0.7131	0.0714	0.0251	0.134*	0.23 (4)
C27	0.8435 (5)	0.0177 (4)	0.2396 (2)	0.1042 (17)	
H27A	0.7813	0.0778	0.2436	0.156*	
H27B	0.9210	0.0432	0.2575	0.156*	
H27C	0.8148	−0.0515	0.2596	0.156*	
C28	0.8640 (4)	−0.0071 (4)	0.1764 (2)	0.0789 (13)	
C29	0.8976 (5)	0.0933 (5)	0.1371 (3)	0.121 (2)	
H29A	0.9781	0.1237	0.1489	0.181*	
H29B	0.8350	0.1531	0.1409	0.181*	
H29C	0.9015	0.0678	0.0960	0.181*	

### Atomic displacement parameters (Å^2^)

**Table:**

	*U*^11^	*U*^22^	*U*^33^	*U*^12^	*U*^13^	*U*^23^
S1	0.0911 (8)	0.0713 (7)	0.0519 (5)	−0.0094 (7)	0.0110 (5)	0.0107 (6)
O1	0.0627 (16)	0.0337 (14)	0.0572 (15)	0.0065 (12)	−0.0024 (12)	−0.0024 (12)
O2	0.0736 (19)	0.0572 (17)	0.0549 (15)	−0.0222 (14)	−0.0119 (13)	0.0024 (14)
O3	0.0572 (15)	0.0353 (14)	0.0430 (13)	−0.0039 (11)	0.0002 (11)	0.0028 (11)
O4	0.0424 (14)	0.0578 (16)	0.0584 (15)	0.0122 (13)	0.0034 (12)	0.0013 (13)
O5	0.0514 (15)	0.0399 (13)	0.0596 (14)	0.0139 (12)	0.0073 (12)	−0.0035 (12)
O6	0.0414 (12)	0.0389 (14)	0.0394 (12)	−0.0081 (11)	0.0005 (10)	−0.0031 (11)
O7	0.0626 (16)	0.0597 (16)	0.0468 (14)	−0.0095 (13)	0.0118 (12)	0.0023 (13)
O8	0.071 (2)	0.070 (2)	0.098 (2)	0.0072 (17)	−0.0062 (16)	−0.0238 (19)
O10	0.0470 (14)	0.0542 (16)	0.0563 (14)	−0.0017 (13)	−0.0092 (11)	0.0032 (13)
C1	0.0373 (18)	0.0317 (18)	0.0382 (17)	0.0015 (15)	−0.0010 (14)	0.0006 (15)
C2	0.041 (2)	0.037 (2)	0.0408 (19)	0.0023 (16)	−0.0052 (15)	−0.0010 (16)
C4	0.047 (2)	0.041 (2)	0.0416 (18)	−0.0012 (17)	0.0037 (15)	0.0045 (17)
C5	0.063 (3)	0.058 (3)	0.048 (2)	−0.011 (2)	0.0114 (18)	0.004 (2)
C6	0.062 (2)	0.071 (3)	0.045 (2)	−0.003 (2)	0.0146 (18)	0.000 (2)
C7	0.057 (2)	0.053 (2)	0.0373 (17)	0.0018 (19)	0.0040 (16)	−0.0011 (17)
C8	0.050 (2)	0.042 (2)	0.0355 (16)	−0.0003 (17)	−0.0017 (15)	0.0006 (15)
C9	0.052 (2)	0.047 (2)	0.0377 (18)	−0.007 (2)	−0.0063 (16)	0.0049 (17)
C11	0.047 (2)	0.0375 (19)	0.0440 (18)	0.0004 (17)	−0.0046 (16)	0.0035 (16)
C12	0.0377 (18)	0.0344 (18)	0.0353 (16)	−0.0008 (15)	0.0000 (14)	0.0002 (14)
C13	0.0366 (18)	0.0321 (17)	0.0357 (16)	−0.0017 (15)	0.0003 (14)	−0.0021 (14)
C14	0.047 (2)	0.0361 (19)	0.0408 (17)	0.0009 (17)	−0.0030 (15)	0.0000 (16)
C15	0.054 (2)	0.036 (2)	0.0459 (19)	0.0022 (18)	−0.0027 (16)	0.0038 (16)
C16	0.0428 (19)	0.038 (2)	0.0388 (17)	−0.0013 (17)	−0.0062 (15)	0.0031 (16)
C17	0.043 (2)	0.042 (2)	0.0399 (17)	−0.0026 (17)	0.0018 (15)	−0.0015 (16)
C18	0.041 (2)	0.0352 (19)	0.0410 (18)	0.0019 (16)	0.0004 (15)	−0.0038 (15)
C19	0.0365 (18)	0.0330 (19)	0.0400 (17)	0.0008 (15)	−0.0032 (14)	−0.0010 (15)
C20	0.058 (2)	0.056 (2)	0.056 (2)	−0.008 (2)	0.0032 (18)	0.0025 (19)
C21	0.086 (3)	0.069 (3)	0.050 (2)	0.012 (2)	0.010 (2)	−0.005 (2)
C22	0.080 (3)	0.071 (3)	0.0380 (19)	0.002 (2)	−0.0034 (18)	0.0052 (19)
C23	0.0373 (19)	0.042 (2)	0.049 (2)	−0.0040 (18)	−0.0053 (16)	0.0023 (18)
C24	0.060 (2)	0.048 (2)	0.056 (2)	−0.0148 (19)	−0.0020 (18)	−0.0074 (18)
C25	0.097 (16)	0.071 (15)	0.063 (13)	−0.018 (11)	0.009 (9)	−0.001 (12)
C26	0.103 (12)	0.084 (11)	0.082 (13)	−0.006 (9)	0.001 (10)	−0.016 (10)
C25'	0.10 (4)	0.07 (4)	0.06 (3)	−0.02 (3)	0.009 (19)	0.00 (3)
C26'	0.10 (2)	0.08 (3)	0.08 (3)	−0.01 (2)	0.00 (2)	−0.02 (2)
C25"	0.10 (4)	0.08 (4)	0.08 (5)	−0.01 (3)	0.00 (3)	−0.02 (5)
C26"	0.10 (3)	0.08 (2)	0.08 (2)	−0.01 (2)	0.00 (2)	−0.016 (17)
C27	0.103 (4)	0.092 (4)	0.118 (4)	0.001 (3)	−0.005 (3)	−0.050 (3)
C28	0.050 (3)	0.065 (3)	0.121 (4)	0.005 (3)	−0.012 (3)	−0.015 (3)
C29	0.100 (4)	0.090 (4)	0.173 (6)	0.013 (4)	0.007 (4)	0.007 (4)

### Geometric parameters (Å, °)

**Table:**

S1—C25	1.80 (4)	C15—H15B	0.9700
S1—C20	1.809 (4)	C16—C19	1.534 (4)
S1—C25"	1.81 (11)	C16—C17	1.543 (4)
S1—C25'	1.82 (5)	C16—H16	0.9800
O1—C2	1.200 (4)	C17—C20	1.506 (4)
O2—C9	1.198 (4)	C17—C18	1.517 (4)
O3—C2	1.339 (4)	C17—H17	0.9800
O3—C4	1.461 (4)	C19—H19	0.9800
O4—C18	1.206 (4)	C20—H20A	0.9700
O5—C14	1.428 (4)	C20—H20B	0.9700
O5—H5	0.8200	C21—H21A	0.9600
O6—C23	1.354 (4)	C21—H21B	0.9600
O6—C19	1.437 (4)	C21—H21C	0.9600
O7—C23	1.196 (4)	C22—H22A	0.9600
O8—C28	1.208 (5)	C22—H22B	0.9600
O10—C9	1.357 (4)	C22—H22C	0.9600
O10—C11	1.441 (4)	C23—C24	1.486 (4)
C1—C2	1.517 (4)	C24—H24A	0.9600
C1—C18	1.528 (4)	C24—H24B	0.9600
C1—C19	1.553 (4)	C24—H24C	0.9600
C1—C13	1.589 (4)	C25—C26	1.54 (5)
C4—C5	1.512 (4)	C25—H25A	0.9700
C4—C12	1.515 (4)	C25—H25B	0.9700
C4—H4	0.9800	C26—H26A	0.9600
C5—C6	1.514 (5)	C26—H26B	0.9600
C5—H5A	0.9700	C26—H26C	0.9600
C5—H5B	0.9700	C25'—C26'	1.50 (9)
C6—C7	1.531 (5)	C25'—H25C	0.9700
C6—H6A	0.9700	C25'—H25D	0.9700
C6—H6B	0.9700	C26'—H26D	0.9600
C7—C21	1.537 (5)	C26'—H26E	0.9600
C7—C22	1.537 (5)	C26'—H26F	0.9600
C7—C8	1.569 (4)	C25"—C26"	1.54 (15)
C8—C9	1.500 (5)	C25"—H25E	0.9700
C8—C12	1.544 (4)	C25"—H25F	0.9700
C8—H8	0.9800	C26"—H26G	0.9600
C11—C12	1.538 (4)	C26"—H26H	0.9600
C11—H11A	0.9700	C26"—H26I	0.9600
C11—H11B	0.9700	C27—C28	1.453 (6)
C12—C13	1.553 (4)	C27—H27A	0.9600
C13—C14	1.523 (4)	C27—H27B	0.9600
C13—H13	0.9800	C27—H27C	0.9600
C14—C15	1.537 (4)	C28—C29	1.491 (7)
C14—H14	0.9800	C29—H29A	0.9600
C15—C16	1.533 (4)	C29—H29B	0.9600
C15—H15A	0.9700	C29—H29C	0.9600
			
C25—S1—C20	103.5 (12)	C20—C17—H17	107.1
C25—S1—C25"	16 (3)	C18—C17—H17	107.1
C20—S1—C25"	115 (4)	C16—C17—H17	107.1
C25—S1—C25'	29 (2)	O4—C18—C17	125.6 (3)
C20—S1—C25'	92 (2)	O4—C18—C1	125.2 (3)
C25"—S1—C25'	28 (4)	C17—C18—C1	109.0 (3)
C2—O3—C4	116.7 (2)	O6—C19—C16	107.2 (2)
C14—O5—H5	109.5	O6—C19—C1	111.4 (2)
C23—O6—C19	116.3 (2)	C16—C19—C1	100.5 (2)
C9—O10—C11	109.9 (3)	O6—C19—H19	112.3
C2—C1—C18	111.2 (3)	C16—C19—H19	112.3
C2—C1—C19	113.3 (3)	C1—C19—H19	112.3
C18—C1—C19	101.8 (2)	C17—C20—S1	113.3 (3)
C2—C1—C13	116.2 (2)	C17—C20—H20A	108.9
C18—C1—C13	103.9 (2)	S1—C20—H20A	108.9
C19—C1—C13	109.1 (2)	C17—C20—H20B	108.9
O1—C2—O3	118.6 (3)	S1—C20—H20B	108.9
O1—C2—C1	122.4 (3)	H20A—C20—H20B	107.7
O3—C2—C1	119.0 (3)	C7—C21—H21A	109.5
O3—C4—C5	107.8 (3)	C7—C21—H21B	109.5
O3—C4—C12	107.0 (2)	H21A—C21—H21B	109.5
C5—C4—C12	114.9 (3)	C7—C21—H21C	109.5
O3—C4—H4	109.0	H21A—C21—H21C	109.5
C5—C4—H4	109.0	H21B—C21—H21C	109.5
C12—C4—H4	109.0	C7—C22—H22A	109.5
C4—C5—C6	107.9 (3)	C7—C22—H22B	109.5
C4—C5—H5A	110.1	H22A—C22—H22B	109.5
C6—C5—H5A	110.1	C7—C22—H22C	109.5
C4—C5—H5B	110.1	H22A—C22—H22C	109.5
C6—C5—H5B	110.1	H22B—C22—H22C	109.5
H5A—C5—H5B	108.4	O7—C23—O6	122.4 (3)
C5—C6—C7	113.0 (3)	O7—C23—C24	127.0 (3)
C5—C6—H6A	109.0	O6—C23—C24	110.5 (3)
C7—C6—H6A	109.0	C23—C24—H24A	109.5
C5—C6—H6B	109.0	C23—C24—H24B	109.5
C7—C6—H6B	109.0	H24A—C24—H24B	109.5
H6A—C6—H6B	107.8	C23—C24—H24C	109.5
C6—C7—C21	109.5 (3)	H24A—C24—H24C	109.5
C6—C7—C22	109.4 (3)	H24B—C24—H24C	109.5
C21—C7—C22	108.1 (3)	C26—C25—S1	106 (2)
C6—C7—C8	108.8 (3)	C26—C25—H25A	110.4
C21—C7—C8	107.1 (3)	S1—C25—H25A	110.4
C22—C7—C8	113.9 (3)	C26—C25—H25B	110.4
C9—C8—C12	103.0 (3)	S1—C25—H25B	110.4
C9—C8—C7	108.9 (3)	H25A—C25—H25B	108.6
C12—C8—C7	116.9 (3)	C25—C26—H26A	109.5
C9—C8—H8	109.2	C25—C26—H26B	109.5
C12—C8—H8	109.2	H26A—C26—H26B	109.5
C7—C8—H8	109.2	C25—C26—H26C	109.5
O2—C9—O10	120.8 (3)	H26A—C26—H26C	109.5
O2—C9—C8	128.8 (3)	H26B—C26—H26C	109.5
O10—C9—C8	110.4 (3)	C26'—C25'—S1	127 (6)
O10—C11—C12	106.0 (2)	C26'—C25'—H25C	105.6
O10—C11—H11A	110.5	S1—C25'—H25C	105.6
C12—C11—H11A	110.5	C26'—C25'—H25D	105.6
O10—C11—H11B	110.5	S1—C25'—H25D	105.6
C12—C11—H11B	110.5	H25C—C25'—H25D	106.1
H11A—C11—H11B	108.7	C25'—C26'—H26D	109.5
C4—C12—C11	112.2 (3)	C25'—C26'—H26E	109.5
C4—C12—C8	115.9 (3)	H26D—C26'—H26E	109.5
C11—C12—C8	100.6 (2)	C25'—C26'—H26F	109.5
C4—C12—C13	107.1 (2)	H26D—C26'—H26F	109.5
C11—C12—C13	107.6 (2)	H26E—C26'—H26F	109.5
C8—C12—C13	113.1 (2)	C26"—C25"—S1	116 (8)
C14—C13—C12	116.6 (2)	C26"—C25"—H25E	108.3
C14—C13—C1	112.3 (2)	S1—C25"—H25E	108.3
C12—C13—C1	109.3 (2)	C26"—C25"—H25F	108.3
C14—C13—H13	105.9	S1—C25"—H25F	108.3
C12—C13—H13	105.9	H25E—C25"—H25F	107.4
C1—C13—H13	105.9	C25"—C26"—H26G	109.5
O5—C14—C13	110.8 (2)	C25"—C26"—H26H	109.5
O5—C14—C15	110.8 (3)	H26G—C26"—H26H	109.5
C13—C14—C15	111.1 (3)	C25"—C26"—H26I	109.5
O5—C14—H14	108.0	H26G—C26"—H26I	109.5
C13—C14—H14	108.0	H26H—C26"—H26I	109.5
C15—C14—H14	108.0	C28—C27—H27A	109.5
C16—C15—C14	114.2 (3)	C28—C27—H27B	109.5
C16—C15—H15A	108.7	H27A—C27—H27B	109.5
C14—C15—H15A	108.7	C28—C27—H27C	109.5
C16—C15—H15B	108.7	H27A—C27—H27C	109.5
C14—C15—H15B	108.7	H27B—C27—H27C	109.5
H15A—C15—H15B	107.6	O8—C28—C27	122.6 (5)
C15—C16—C19	108.2 (2)	O8—C28—C29	120.4 (5)
C15—C16—C17	115.2 (3)	C27—C28—C29	117.0 (5)
C19—C16—C17	102.3 (3)	C28—C29—H29A	109.5
C15—C16—H16	110.3	C28—C29—H29B	109.5
C19—C16—H16	110.3	H29A—C29—H29B	109.5
C17—C16—H16	110.3	C28—C29—H29C	109.5
C20—C17—C18	111.1 (3)	H29A—C29—H29C	109.5
C20—C17—C16	120.9 (3)	H29B—C29—H29C	109.5
C18—C17—C16	102.9 (2)		
			
C4—O3—C2—O1	179.3 (3)	C2—C1—C13—C12	−7.7 (4)
C4—O3—C2—C1	−0.1 (4)	C18—C1—C13—C12	−130.2 (3)
C18—C1—C2—O1	−29.1 (4)	C19—C1—C13—C12	121.8 (3)
C19—C1—C2—O1	84.8 (4)	C12—C13—C14—O5	−49.6 (3)
C13—C1—C2—O1	−147.7 (3)	C1—C13—C14—O5	77.6 (3)
C18—C1—C2—O3	150.3 (3)	C12—C13—C14—C15	−173.2 (3)
C19—C1—C2—O3	−95.8 (3)	C1—C13—C14—C15	−45.9 (4)
C13—C1—C2—O3	31.7 (4)	O5—C14—C15—C16	−81.9 (3)
C2—O3—C4—C5	−177.2 (3)	C13—C14—C15—C16	41.6 (4)
C2—O3—C4—C12	−53.1 (3)	C14—C15—C16—C19	19.2 (4)
O3—C4—C5—C6	173.9 (3)	C14—C15—C16—C17	−94.5 (3)
C12—C4—C5—C6	54.7 (4)	C15—C16—C17—C20	−43.5 (4)
C4—C5—C6—C7	−65.9 (4)	C19—C16—C17—C20	−160.6 (3)
C5—C6—C7—C21	174.2 (3)	C15—C16—C17—C18	81.1 (3)
C5—C6—C7—C22	−67.5 (4)	C19—C16—C17—C18	−36.0 (3)
C5—C6—C7—C8	57.4 (4)	C20—C17—C18—O4	−34.6 (4)
C6—C7—C8—C9	−155.1 (3)	C16—C17—C18—O4	−165.3 (3)
C21—C7—C8—C9	86.6 (4)	C20—C17—C18—C1	140.5 (3)
C22—C7—C8—C9	−32.8 (4)	C16—C17—C18—C1	9.8 (3)
C6—C7—C8—C12	−39.0 (4)	C2—C1—C18—O4	−44.2 (4)
C21—C7—C8—C12	−157.2 (3)	C19—C1—C18—O4	−165.2 (3)
C22—C7—C8—C12	83.3 (4)	C13—C1—C18—O4	81.5 (4)
C11—O10—C9—O2	178.5 (3)	C2—C1—C18—C17	140.7 (3)
C11—O10—C9—C8	0.5 (3)	C19—C1—C18—C17	19.7 (3)
C12—C8—C9—O2	162.4 (3)	C13—C1—C18—C17	−93.7 (3)
C7—C8—C9—O2	−72.9 (4)	C23—O6—C19—C16	−163.2 (2)
C12—C8—C9—O10	−19.8 (3)	C23—O6—C19—C1	87.8 (3)
C7—C8—C9—O10	104.9 (3)	C15—C16—C19—O6	169.9 (2)
C9—O10—C11—C12	19.4 (3)	C17—C16—C19—O6	−68.1 (3)
O3—C4—C12—C11	−43.7 (3)	C15—C16—C19—C1	−73.6 (3)
C5—C4—C12—C11	76.0 (4)	C17—C16—C19—C1	48.4 (3)
O3—C4—C12—C8	−158.5 (2)	C2—C1—C19—O6	−47.4 (3)
C5—C4—C12—C8	−38.9 (4)	C18—C1—C19—O6	72.0 (3)
O3—C4—C12—C13	74.2 (3)	C13—C1—C19—O6	−178.6 (2)
C5—C4—C12—C13	−166.2 (3)	C2—C1—C19—C16	−160.8 (3)
O10—C11—C12—C4	−153.8 (2)	C18—C1—C19—C16	−41.3 (3)
O10—C11—C12—C8	−29.9 (3)	C13—C1—C19—C16	68.1 (3)
O10—C11—C12—C13	88.6 (3)	C18—C17—C20—S1	178.0 (2)
C9—C8—C12—C4	150.4 (3)	C16—C17—C20—S1	−61.4 (4)
C7—C8—C12—C4	31.1 (4)	C25—S1—C20—C17	−149.1 (14)
C9—C8—C12—C11	29.2 (3)	C25"—S1—C20—C17	−138 (4)
C7—C8—C12—C11	−90.2 (3)	C25'—S1—C20—C17	−122 (2)
C9—C8—C12—C13	−85.3 (3)	C19—O6—C23—O7	−1.4 (4)
C7—C8—C12—C13	155.3 (3)	C19—O6—C23—C24	179.5 (2)
C4—C12—C13—C14	87.3 (3)	C20—S1—C25—C26	84 (2)
C11—C12—C13—C14	−151.8 (3)	C25"—S1—C25—C26	−57 (21)
C8—C12—C13—C14	−41.6 (4)	C25'—S1—C25—C26	14 (5)
C4—C12—C13—C1	−41.3 (3)	C25—S1—C25'—C26'	−63 (6)
C11—C12—C13—C1	79.6 (3)	C20—S1—C25'—C26'	−177 (6)
C8—C12—C13—C1	−170.2 (2)	C25"—S1—C25'—C26'	−29 (7)
C2—C1—C13—C14	−138.8 (3)	C25—S1—C25"—C26"	63 (19)
C18—C1—C13—C14	98.8 (3)	C20—S1—C25"—C26"	20 (8)
C19—C1—C13—C14	−9.2 (3)	C25'—S1—C25"—C26"	−16 (6)

### Hydrogen-bond geometry (Å, °)

**Table:**

*D*—H···*A*	*D*—H	H···*A*	*D*···*A*	*D*—H···*A*
O5—H5···O8^i^	0.82	2.16	2.952 (4)	161

Symmetry codes: (i) −*x*+1, *y*+1/2, −*z*+1/2.
